# Influence of Different Types of Polysaccharide-Based Coatings on the Storage Stability of Fresh-Cut Kiwi Fruit: Assessing the Physicochemical, Antioxidant and Phytochemical Properties

**DOI:** 10.3390/foods10112806

**Published:** 2021-11-15

**Authors:** Ishrat Guroo, Amir Gull, Sajad Mohd Wani, Sajad Ahmad Wani, Asma A. Al-Huqail, Jwaher Haji Alhaji

**Affiliations:** 1 Department of Food Science and Technology, University of Kashmir, Hazratbal, Srinagar 190006, India; ishrat12@gmail.com; 2 Division of Food Science and Technology, Sher-e-Kashmir University of Science and Technology, Shalimar 190025, India; dramirft@gmail.com; 3 Department of Food Technology, Islamic University of Science and Technology, Awantipora 192122, India; sajad04@outlook.com; 4 Department of Botany and Microbiology, College of Science, King Saud University, Riyadh 11451, Saudi Arabia; aalhuqail@ksu.edu.sa; 5 Department of Health Science, College of Applied Studies and Community Service, King Saud University, Riyadh 11451, Saudi Arabia; jalhejjy@ksu.edu.sa

**Keywords:** kiwi fruit, edible coatings, storage, total phenolics, antioxidants

## Abstract

The present study focuses on studying the influence of various edible biopolymer coatings at several concentrations on physicochemical, antioxidant and lipid peroxidation activity levels of biopolymer-coated fresh-cut kiwi slices stored at room temperature (relative humidity: 90%). Kiwi slices were coated by dipping in xanthan gum (0.1, 0.2, 0.3% *w*/*v*), alginate (1, 2, 3% *w*/*v*) and chitosan (0.25, 0.50, 0.75% *w*/*v*) solutions for 2 min. Kiwi fruit slices without any treatment were designated as the control. Compared to the control, all coated samples retained higher ascorbic acid, titratable acidity, total phenolic component and antioxidant capacity levels. However, xanthan-gum-coated slices retained significantly higher amounts of total phenolics in comparison to alginate- and chitosan-coated slices (*p* ≤ 0.05). HPLC analysis showed the presence of neochlorogenic acid, chlorogenic acid, ellagic acid and epicatechin. The results suggest that the xanthan gum can be utilized to enhance the shelf life of fresh-cut kiwi slices without compromising quality.

## 1. Introduction

Fresh-cut fruit should retain its fresh attributes throughout its shelf life. However, during preparation, serious damage to tissues can occur due to peeling and slicing. This damage can result in changes in color, loss of texture and oxidation of vitamins, in addition to other undesirable biochemical reactions. Further, the leakage of fruit juices provides nutrients to the microbes, stimulating their growth, which may lead to foodborne illness [1,2]. Therefore, the shelf life of fresh-cut fruit is constrained to just a couple of days. Extending the shelf life may be a considerable challenge. Kiwi is sensitive to ethylene since it is a climacteric fruit, which is liked due to its flavor and ascorbic acid content [3]. In the past few years, the interest in fresh-cut kiwi has expanded significantly, since awareness among consumers regarding healthy eating habits has increased. However, as already discussed above, serious damage can occur in fresh-cut kiwi slices during preparation, thereby minimizing their shelf life and quality. Since fresh-cut kiwis are stored before being consumed, storage-induced changes are inevitable. Vitamins and other nutrients are lost during storage [4]. It is, therefore, of foremost importance to find better and more effective techniques to preserve these fresh-cut kiwi fruits. Various methods, including the use of chemical and natural preservatives, active packaging and edible coatings, have been employed to enhance the shelf life of fresh-cut kiwi slices; however, use of edible coatings is preferred. This is because some edible hydrocolloid-based coatings exhibit antioxidant potential that helps to retain the fruit color.

Edible coatings could be applied to fresh-cut kiwi to prevent storage-induced changes from occurring. The demand for these edible coatings has increased considerably during recent times to growing concerns over the effects of plastics on the environment [5]. These coatings have been widely studied for their ability to prolong the shelf life of fruits by minimizing water loss and fungal growth [6]. Active ingredients such as flavorants, antioxidant components, bioactive components and antimicrobial agents could be incorporated into coatings [7]. Xanthan gum, a polysaccharide produced by *Xanthomonas*, has been reported to decrease losses in weight and oxidative browning in fresh-cut apple [8]. Alginate is extracted from phaeophyceae and is comprised of *β*-D-mannuronic acid and *α*-L-guluronic acid. Its film-forming behavior is due to its capacity to combine with di- and trivalent cations such as calcium and magnesium, which are added as gelling agents. Diaz-Mula et al. (2012) [9] and Mastromatteo et al. (2011) [10] observed that alginate-based coatings maintained fruit quality by reducing the respiratory rate and delayed dehydration in sweet cherry and kiwi fruit slices, respectively. Chitosan is obtained either directly from fungi or via chemical deacetylation [11]. It can be used as surface coating material for fruits, since it possesses excellent film-forming properties. Chitosan coatings delay the ripening of several fruits [12]. Chitosan has received considerable attention due to it being biodegradable, biocompatible, non-toxic, environmentally friendly and possessing antimicrobial activity [13]. This linear polysaccharide has been extensively employed in edible coatings to retain the quality of produce.

As far as we know, no or very few studies have been carried out on the ambient storage-induced changes in physicochemical and antioxidant characteristics of kiwi fruit. Therefore, this study was undertaken with the aim of studying in detail the changes that occur in physicochemical, antioxidant and lipid peroxidation activity in fresh-cut kiwi slices stored at room temperature and high relative humidity.

## 2. Materials and Methods

### 2.1. Materials

Kiwi samples (*Actinidia deliciosa* cultivar Hayward) at physiological maturity were obtained from local vegetable vendors in Hazratbal Srinagar, Jammu and Kashmir, India. The fruits were selected based on uniformity of shape and size—damaged and defective fruits were discarded. Food-grade biopolymers such as sodium alginate powder (AL), chitosan (CH) (95% deacetylated) and xanthan gum (XG) were purchased from HIMEDIA. (L.B.S. Marg, Mumbai, India.) Calcium lactate was purchased from Sigma-Aldrich (St. Louis, MO, USA).

#### 2.1.1. Preparation of Coatings

Coatings were synthesized by dissolving sodium alginate (AL) (1, 2 and 3% *w*/*v*) and xanthan gum (XG) (0.1, 0.2 and 0.3% *w*/*v*) in distilled water at 60 °C with continuous stirring using a magnetic stirrer (T25 digital Ultra-Turrax mixer-IKA) at 6500 rpm for 2 h. Chitosan emulsions were made by dissolving CH (0.25, 0.50 and 0.75% *w/v*) in (1% *v*/*v*) glacial acetic acid stirred with a homogenizer at 700× *g* for 5 min The pH of the solution was adjusted to 5.2 with (0.1 M) NaOH. Calcium lactate (2% *w*/*v*) was then added to each coating in order to enhance the polymer cross-linking for better efficacy of edible coatings over fruit.

#### 2.1.2. Preparation of Fruit

Kiwi fruit were sanitized with 1% sodium hypochlorite, then rinsed with tap water and drained. Prior to further processing, excess water was removed using adsorbent paper. Washed fruit were peeled with a knife and sliced with a stainless steel slicer (Bajaj Processpack Ltd., Noida, India) into slices measuring 6 mm thick.

#### 2.1.3. Coating of Fresh-cut Kiwi Slices

The prepared kiwi slices were dipped into sodium alginate (1, 2 and 3% *w*/*v*), xanthan gum (0.1, 0.2 and 0.3% *w*/*v*) and chitosan (0.25, 0.50 and 0.75% *w*/*v*) coating formulations for 2 min, whereas kiwi slices without any treatment were kept as controls (uncoated).

#### 2.1.4. Storage of Coated Fresh-Cut Kiwi Slices

The coatings were dried at room temperature (20 ± 1.0 °C) and kept in polypropylene trays (14 × 9 × 7 cm^3^), then stored at ambient temperature with 80–90% relative humidity. Finally, samples were analyzed at an interval of 2 days until 10 days of storage for certain parameters.

### 2.2. Physicochemical Properties

Weight loss was estimated using the following equation:

(1) $$Weight\;Loss\;(\%)=\frac{Initial\;weight-Weight\;of\;sample}{Initial\;weight}\times 100$$

Total soluble solids were estimated using a hand-held refractrometer (Atago Co., Tokyo, Japan). The TSS analysis was done in triplicate and the results are expressed as percentages, while the titratable acidity was determined using the titration method. The percentage of titratable acidity was calculated using Equation (2):

(2) $$(\%)\;Acidity=\frac{Titer\;value\times Normality\;of\;NaOH\times Volume\;made\times Eqivalent\;weight\;of\;acid\times 100}{Weight\;of\;sample\times Volume\;of\;aliquot\times 1000}\;$$

The dye method was used for the determination of ascorbic acid [14]. Briefly, 10 g of fruit pulp was homogenized with 90 mL of 3% metaphosphoric acid, filtered, and titrated against 2,6-dichlorophenol-indophenol to an end point until a pink color persisted. The ascorbic acid content was calculated using Equation (3):

(3) $$\begin{array}{ll} & Dye\;factor=\frac{0.5}{Titre\;value}\\ mg\;Ascorbic\;acid & /100\;mg\;or\;mL\\ & =\frac{\left(Titre\;Value\times Dye\;factor\times Volume\;made\times 100\right)}{\left(Aliquot\;of\;sample\times Volume\;of\;sample\right)} \end{array}$$

### 2.3. Total Phenolic Content

The total phenolic content (mg GAE/g dry weight) was evaluated via Folin–Ciocalteau assay. Briefly, 50 mL distilled water was added to 50 mg dried extract and dissolved. After this, 0.5 mL Folin–Ciocalteau reagent and 2 mL of 20% NaHCO_3_ were added to the mixture. The reaction mixture was mixed thoroughly and incubated at room temperature for 2 h and its absorbance was measured at 765 nm using a UV-VIS spectrophotometer (HITACHI U-2900) (Chiyoda City, Tokyo, Japan). Total phenolic contents were estimated from the gallic acid calibration curve.

### 2.4. Antioxidant Properties

#### 2.4.1. 2,2-Azino-bis-3-ethylbenzothiazoline-6-sulfonic Acid (ABTS) Scavenging Activity

The ABTS scavenging activity was evaluated following the protocols used by Wei and Gao (2016) [15] with slight modifications. Here, 2.5 mL of ABTS^+^ radical stock solution containing 7.4 mM of ABTS and 2.6 mM of potassium persulfate, which was diluted 12-fold, was added to 100 mL of sample and kept in the dark for 10 min at room temperature to allow the mixture to react. The ABTS^+^ scavenging activity was then calculated at 734 nm using Equation (4):

(4) $$Inhibition\;\left(\%\right)=\frac{\left(A_{\mathrm{control}}-A_{\mathrm{sample}}\right)}{\left(A_{\mathrm{control}}\right)}\times 100$$

where A_control_ represents the absorbance of the control and A_sample_ represents the absorbance of the sample.

#### 2.4.2. 2,2-Diphenyl-1-picrylhydrazyl (DPPH) Radical Scavenging Activity

The DPPH free radical scavenging activity was estimated following the protocols used by Brand-Williams et al. (1995) [16], with slight modifications. Briefly, 10 mL of methanol was mixed with 10 mg of sample, then 2 mL of 60 mM DPPH in methanol was added. The solution was then placed in the dark for 30 min. The percentage of inhibition was then calculated at 517 nm using Equation (4), where A_control_ represents the absorbance of the control and A_sample_ represents the absorbance of the sample.

### 2.5. Lipid Peroxidation Assay

The lipid peroxidation assay was carried out following the protocols used by Wright et al. (1981) [17]. Here, 1 mL of linoleic acid, 0.2 mL of ferric nitrate (20 mM), 0.2 mL of ascorbic acid (200 mM), 0.2 mL of H_2_O_2_, and different concentrations of extract (100 µL) were mixed to a total volume of 2 mL followed by incubation at 37 °C in a water bath for 1 h. About 1 mL of trichloro acetic acid (10% *w*/*v*) was added followed by 1 mL of 1% TBA, then the tubes were kept in a boiling water bath for 20 min. The tubes were then centrifuged for 10 min at 5000× *g*. The percentage of inhibition of malonaldehyde was estimated at 535 nm using Equation (4), where A_control_ represents the absorbance of the control and A_sample_ represents absorbance of the sample.

### 2.6. Quantification of Phenolic and Flavonoid Compounds by HPLC

#### 2.6.1. Extraction

Briefly, 50 g kiwi fruit was extracted three times with 80% methanol using a sample-to-solution ratio of 1:2. The extracts obtained after filtration were pooled together and concentrated under reduced pressure at temperatures not exceeding 40 °C using a rotary vacuum evaporator. This concentrated extract was designated as a whole concentrate (WC). The known weight of the sample from the whole extract was extracted three times for phenolics using ethyl acetate and diethyl ether. The ethyl acetate and diethyl ether extracts were combined, passed over anhydrous sodium sulfate for 30 min, and filtered through Whatman filter paper no. 42. The combined ethyl acetate and diethyl ether extracts were then evaporated using a rotary vacuum evaporator and stored in a desiccator prior to analysis by HPLC. The dried sample was redissolved in 1 mL of HPLC-grade methanol before it was injected into HPLC.

#### 2.6.2. Analysis by HPLC

For the quantification and identification of phenolic compounds, the protocols of Wani et al. (2017) [18] were used. Briefly, dried aqueous methanol extracts of all samples were dissolved in HPLC-grade methanol and a 1.0% solution (*w*/*v*) was prepared. A 20 µL aliquot was injected into a C-18 column (250 mm × 4.6 mm) with a diameter of 4.6 mm in an HPLC system (Jasco Corporation, Tokyo, Japan) equipped with a UV detector. The flow rate was maintained at 1.0 mL/min. The elution gradients used to separate the phytochemicals were distilled water, acetonitrile, orthophosphoric acid, acetic acid, and methanol. In order to identify the phenolic compounds, a detector was set at wavelengths of 280 and 320 nm. Identification of the phenolic compounds was done by comparing the retention times (Rt) with standards such as neochlorogenic acid, chlorogenic acid, ellagic acid, epicatechin, phloridzin, kaempferol, Quercetin-3-glucoside, and procyanidin b_2_.

### 2.7. Statistical Analysis

All of the experiments were carried out in triplicate and the results are expressed as mean values. The significant differences were obtained by one-way analysis of variance (ANOVA) followed by Duncan’s multiple range test (*p* < 0.05) using Statistica V.7.

## 3. Results

### 3.1. Weight Loss, TSS, Titratable Acidity, and Ascorbic Acid

Weight loss affects fruit quality during storage and occurs due to transpiration. The results revealed that weight loss increased in both the coated and control samples (CL) during storage, although control sample showed significantly higher weight loss (19.95%) (Table 1). Weight loss also varied depending on the coating and its concentration. Samples coated with XG 0.3%, CH 0.75% and AL 3% exhibited lower weight loss of 10.34%, 11.73% and 10.75%, respectively, amongst which XG 0.3% had a better influence in terms of delaying weight loss, which could be because coatings act as barriers against evaporative water loss by forming a thick layer around the fruit surfaces. Earlier, Vivek and Subbarao (2018) [19] also reported reductions in weight loss for kiwi slices due to the application of chitosan coatings. Reductions in weight loss of plums coated with alginate were reported by Valero et al. (2013) [20].

The effects of edible coatings on the TSS are given in Table 1. The results revealed increases in TSS up to 8 days of storage, after which it decreased. However, the control sample exhibited significantly high TSS (29.86 °B) values compared to the coated samples at the end of the 10th day of storage. Among the coatings, XG 0.3%, CH 0.75% and AL 3% showed significantly lower TSS values of 23.58, 24.33 and 23.73, respectively, which may have been due to the improved oxygen barrier properties of the coatings reducing the respiration rate and ripening process. It was observed that coating treatments maintained the TSS values of slices, which could be due to their excellent semipermeable properties [21].

The TSS was observed to be 29.86 °B in the control as compared to the coated samples. The starch breakdown, hydrolysis of cell wall polysaccharides and increases in dry matter due to water loss may have resulted in increases in TSS [22,23]. Malmiri et al. (2011) [24] also observed a reduction in cellulose-based coated banana slices. Robledo et al. (2018) [25], however, reported that biocoatings did not alter quality parameters such as TSS. Reductions in TSS with gums were also reported by Robles-Flores et al. (2018) [26].

The effects of coatings on titratable acidity are shown in Table 1. The results showed that the titratable acidity decreased in both control and coated kiwi slices throughout the storage period. However, the amount the values decreased varied depending on the coating and concentration. On the 10th day of storage, lower titratable acidity values were reported in the control (0.91%), while the highest value was observed in the sample coated with XG 0.3% (1.81%), followed by AL 3% (1.75%) and CH 0.75% (1.73%). The coatings act as a barrier, thereby reducing the ripening rate, meaning the use of organic acids is delayed [23]. Decreased acidity in papaya fruit coated with chitosan has also been demonstrated by Ali et al. (2011) [27].

Naturally occurring antioxidants (e.g., ascorbic acid) prevent or retard the damage caused by reactive oxygen species (ROS), although after ripening this begins to decline. During storage, the ascorbic acid contents decreased in both control and coated samples from 34.83 to 10.26 mg/100 g FW and 52.8 to 11.93 mg/100 g FW, respectively. However, lower reductions were observed in coated samples. As samples coated with XG 0.3%, XG 0.2%, CH 0.75% and AL 3% showed high ascorbic acid values (32.5 mg/100 g FW, 25.66 mg/100 g FW, 25.83 mg/100 g FW and 24.63 mg/100 g FW, respectively) at the end of storage period, this showed that edible coatings prevented decreases in ascorbic acid content, although non-significantly, which could be ascribed to their oxygen barrier characteristics [28]. Retention of the ascorbic acid content was reported in red kiwi fruit coated with chitosan by [29] Kaya et al. (2016). Simarly, Eltoum and Babiker (2014) [30] and Ali et al. (2011) [27] reported similar results for Arabic- gum-coated tomato and chitosan-coated papaya slices. Silva et al. (2014) [31] also observed the retention of ascorbic acid in pineapple slices coated with pectin.

### 3.2. Total Phenolic Content

The results showed significant increases in total phenolics in coated and control samples up to the 8th day of storage, after which the levels decreased (Table 2). This decrease in phenolics towards the end of the storage period may have been because of fruit senescence and cell structure breakdown [27]. Total phenols were retained at much higher levels in coated kiwi slices than control slices. Kiwi slices coated with XG 0.1%, AL 1% and CH 0.75% showed high phenolic content levels at the end of storage period. Amongst the coatings, the XG 0.1% treatment resulted in the highest phenolic content (0.88 mg GAE/g DW) at the 10th day of storage. This retention of phenolics compounds maybe have been due to the protective barrier properties of the coatings on fruit surfaces, meaning the supply of oxygen was limited during the enzymatic oxidation of phenols. Earlier, Kaya et al. (2016) [29] reported on the retention of total phenols via the application of chitosan coatings on red kiwi fruit. Similarly, Ghasemnezhad et al. (2010) [32] and Ghasemnezhad et al. (2011) [33] reported similar results for apricot and loquat fruit coated with chitosan, xanthan gum and alginate edible coatings.

### 3.3. Antioxidant Activity

The influence of edible coatings on the antioxidant activity of kiwi fruit slices during storage was assessed via the ABTS and DPPH scavenging activity levels. The concentrations of antioxidants in coated and control samples increased up to the 8th day of storage, after which they decreased (Table 2). However, samples coated with XG 0.1%, AL 1% and CH 0.75% showed high antioxidant activity levels of 74.30, 73.86 and 73.86%, respectively, as compared to the control at 61.01% at the end of the 10th day of storage. Among the coatings, XG 0.1%-coated samples showed a high antioxidant capacity level of 71.91% at the end of the storage period. The retention of antioxidant activity caused by edible coatings (Arabic gum) was reported by Khaliq et al. (2016) [34] in mango slices. Similarly, the antioxidant activity levels of coated and control samples as evaluated by DPPH scavenging activity showed increases in radical scavenging activity up to the 8th day of storage, which decreased thereafter. Additionally, the scavenging activity levels varied with different concentrations of coating materials, as XG 0.1%-, AL 1%- and CH 0.75%-coated samples exhibited high scavenging activity levels of 90.82, 90.89 and 92.18%, respectively. It was observed that in comparison to the control, the edible coatings retained their DPPH radical scavenging activity (Table 2). Khaliq et al. (2016) [34] and Addai et al. (2013) [35] also observed increases in scavenging activity for mango and papaya fruit coated with Arabic gum.

Most of the postharvest treatments changed the natural conditions of the fruit so as to extend the postharvest life of the produce. These postharvest treatments affected the metabolic activity of the coated produce by activating the antioxidant system [36]. This activation happens in response to postharvest stress, which is considered to be beneficial as it ameliorates the antioxidant potential of tropical fruits. 

### 3.4. Lipid Peroxidation Assay—Melondialdehyde Content

The lipid peroxidation assay was performed to evaluate the inhibition of malondialdehyde (MDA). Significant (*p* ≤ 0.05) increases in lipid peroxidation were observed up to 8 days of storage, which declined thereafter. Inhibition levels also varied with the type of coating and concentration. In conclusion, the samples coated with biopolymers had higher capacity to inhibit MDA than control. Amongst the coatings used, XG 0.1% resulted in higher inhibition activity of 44.63%.

The results indicate that the xanthan gum could be a promising polysaccharide for preventing postharvest oxidative damage during ambient storage. These coatings prevent lipid peroxidation by acting as a barrier to oxygen, meaning the integrity of the membrane is maintained [37]. Khaliq et al. (2016) [34] observed similar findings for guava and mango coated with chitosan and Arabic gum.

### 3.5. Analysis of Phenolic and Flavonoid Compounds

The effects of various types and concentrations of edible coatings on the phenolic and flavonoid compositions of fresh-cut kiwi fruit are presented in Table 3 and Table 4. HPLC analysis of coated kiwi fruit slices showed the presence of eight compounds—three phenolic acids and five flavonoids. The phenolic acids included ellagic acid and the two hydroxycinnamic acid derivatives chlorogenic acid and neochlorogenic acid, whereas the flavonoids included epicatechin, kaempferol, quercetin-3-glucoside, procyanidin b_2_ and a dihydrochalcone–phloridzin. Among the phenolic acids, neochlorogenic acid and chlorogenic acid were observed to be in abundance in all coated and control samples as compared to ellagic acid. Amongst the flavonoids, the most abundant acid found in coated and control samples was epicatechin, while kaempferol was found to be present in the lowest amounts in these samples.

**Figure 1:**
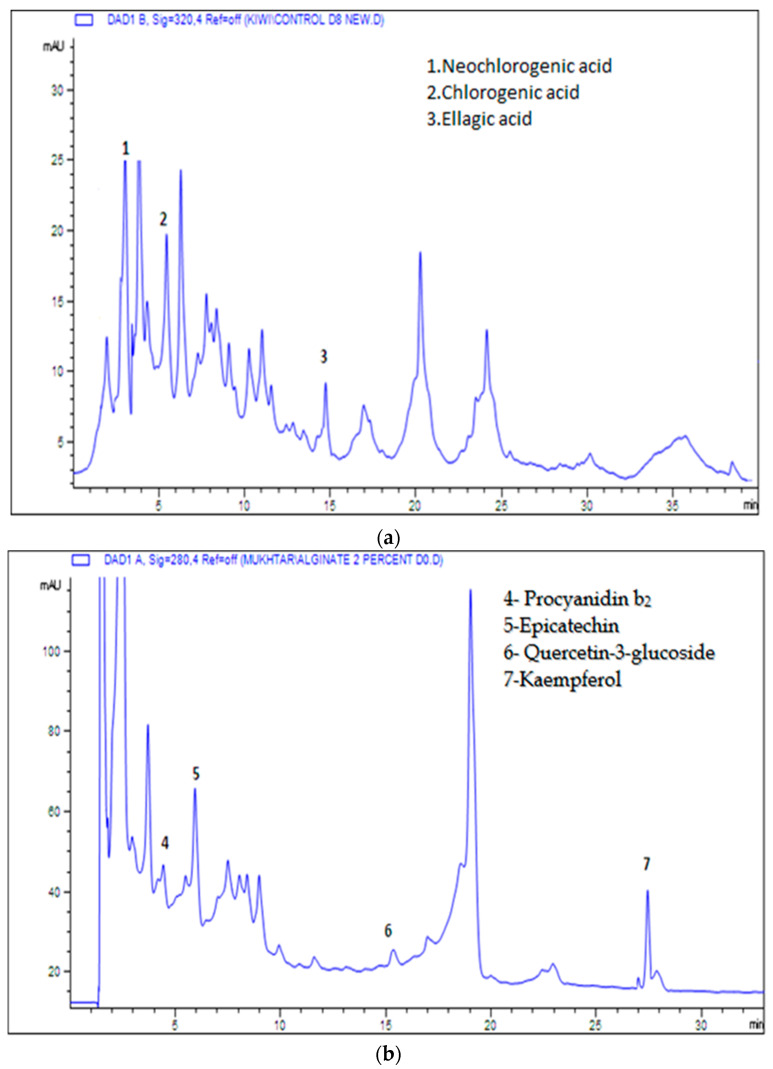
(**a**) Representative HPLC chromatogram of kiwi fruit slices showing polyphenols at 320 nm and 280 nm. (**b**) Representative HPLC chromatogram of kiwi fruit showing polyphenols at 280 nm.

Abiotic stress on fruits is thought to be caused by edible coatings, which modify their metabolism and influence the production of phenolic and flavonoid chemicals (secondary metabolites) [36]. These coatings result in the accumulation of phenolics and ascorbic acid, as indicated already, thereby causing increases in antioxidant activity (Table 2) [37], supporting our results showing increased antioxidant activity in coated samples. Representative HPLC chromatograms of kiwi fruit slices showing polyphenols at 320 nm and 280 nm are shown in Figure 1a,b.

### 3.6. Appearance Changes during Storage

**Figure 2:**
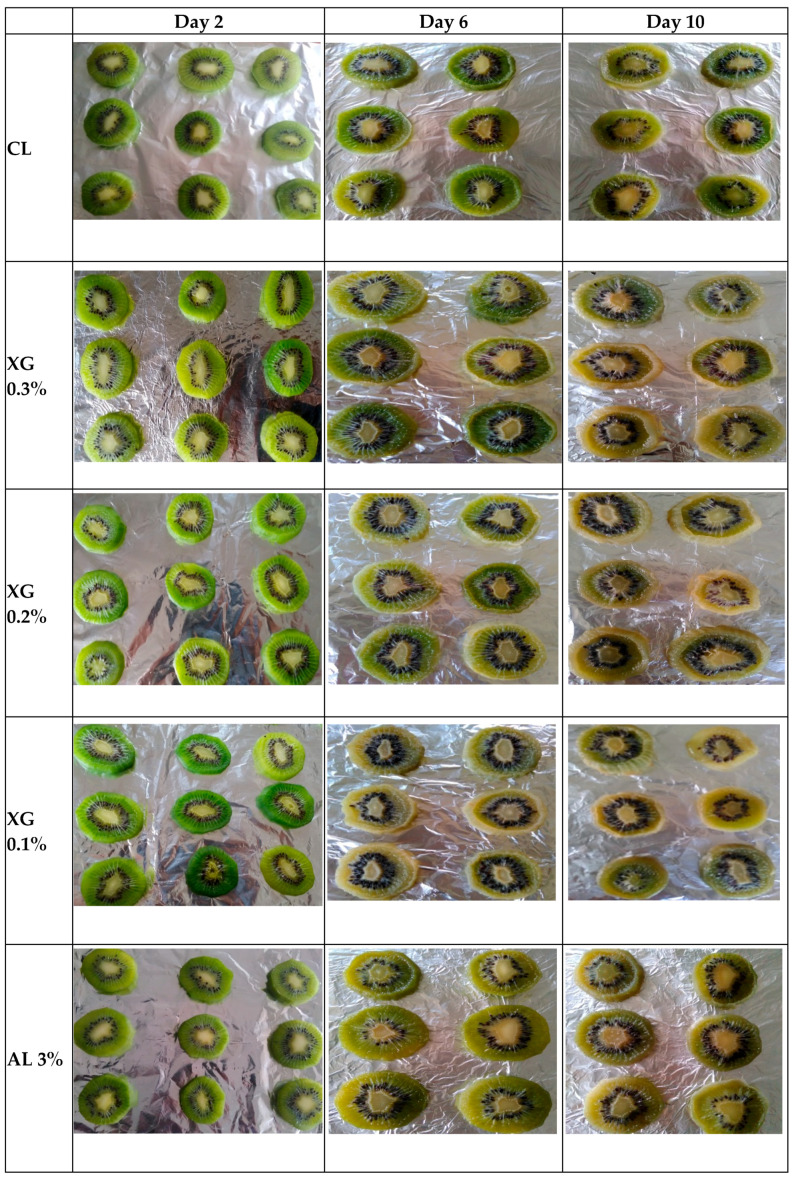

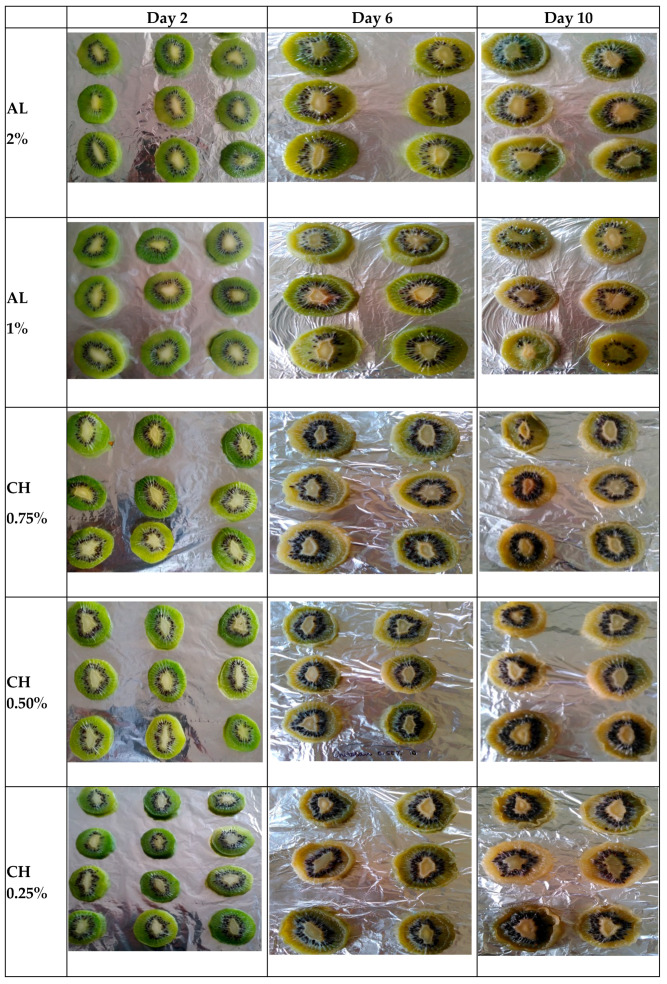
Changes in the appearance of polysaccharide-based coated kiwi fruit slices during storage.

Changes in the appearance of polysaccharide-based coated kiwi fruit slices during storage are shown in Figure 2.

## 4. Conclusions

This study indicated that not all edible coatings effectively maintained the quality of fresh-cut kiwi slices fruit during storage. However, xanthan was found to have a better preserving influence on the physicochemical and antioxidant characteristics of kiwi slices, in addition to providing better inhibitory action against MDA. The chemical analysis also reflected the higher amounts of phenolics in xanthan-gum-coated kiwi slices. The use of xanthan gum also resulted in higher levels of antioxidants, as evident from higher the DPPH radical scavenging activity levels. Changes in TSS, titratable acidity and ascorbic acid levels further revealed that the use of xanthan gum (0.3%) resulted in lower decomposition rates. To summarize, this study suggests that xanthan gum could be useful for maintaining the quality and enhancing the storage life of fresh-cut kiwi slices.

## Figures and Tables

**Table 1:** Effects of polysaccharide-based coatings and storage on weight loss (%), total soluble solids (°Brix), titratable acidity values (% acidity) and ascorbic acid contents (mg/100 g FW).

Treatment	Days of Storage				
	2	4	6	8	10
(A) Weight loss (%)					
CL	10.03 ± 0.03 ^aE^	12.18 ± 0.02 ^aD^	15.12 ± 0.04 ^aC^	18.42 ± 0.03 ^aB^	19.95 ± 0.03 ^aA^
AL 1%	9.55 ± 0.04 ^bE^	10.72 ± 0.03 ^bD^	12.88 ± 0.04 ^cC^	15.51 ± 0.05 ^bB^	17.58 ± 0.04 ^bA^
AL 2%	7.23 ± 0.05 ^dE^	8.29 ± 0.05 ^dD^	9.25 ± 0.02 ^fC^	10.39 ± 0.01 ^hB^	12.52 ± 0.04 ^fA^
AL 3%	6.22 ± 0.03 ^eE^	7.46 ± 0.08 ^eD^	8.61 ± 0.05 ^gC^	9.71 ± 0.06 ^iB^	10.75 ± 0.04 ^hA^
CH 0.25%	9.43 ± 0.07 ^bE^	10.77 ± 0.05 ^bD^	13.24 ± 0.04 ^bC^	14.96 ± 0.05 ^cB^	16.68 ± 0.03 ^cA^
CH 0.50%	8.29 ± 0.02 ^cE^	9.43 ± 0.01 ^cD^	11.39 ± 0.03 ^dC^	12.76 ± 0.03 ^eB^	13.74 ± 0.05 ^eA^
CH 0.75%	7.32 ± 0.08 ^dE^	8.82 ± 0.05 ^dD^	10.53 ±0.07 ^eC^	11.01 ± 0.04 ^gB^	11.73 ± 0.05 ^gA^
XG 0.1%	7.44 ± 0.09 ^dE^	8.65 ± 0.05 ^dD^	11.62 ± 0.03 ^dC^	13.08 ± 0.07 ^dB^	14.18 ± 0.08 ^dA^
XG 0.2%	6.28 ± 0.07 ^eE^	7.62 ± 0.05 ^eD^	9.98 ± 0.04 ^fC^	11.35 ± 0.02 ^fB^	12.38 ± 0.04 ^fA^
XG 0.3%	5.47 ± 0.02 ^fE^	6.84 ± 0.09 ^fD^	8.46 ± 0.03 ^gC^	9.53 ± 0.05 ^iB^	10.34 ± 0.02 ^hA^
(B) Total soluble solids (°Brix)					
CL	26.13 ± 0.09 ^aE^	27.2 ± 0.02 ^aD^	28.13 ± 0.25 ^aC^	29.86 ± 0.19 ^aA^	28.13 ± 0.09 ^aB^
AL 1%	25.06 ± 0.09 ^bE^	25.46 ± 0.09 ^bD^	26.27 ± 0.25 ^bC^	26.93 ± 0.09 ^bA^	26.06 ± 0.07 ^bB^
AL 2%	24.06 ± 0.07 ^cE^	24.33 ± 0.08 ^cD^	25.13 ± 0.19 ^dB^	25.33 ± 0.09 ^cA^	24.46 ± 0.38 ^dC^
AL 3%	23.13 ± 0.18 ^dE^	23.4 ± 0.12 ^dD^	23.93 ± 0.09 ^fB^	24.80 ± 0.32 ^dA^	23.73 ± 0.18 ^eC^
CH 0.25%	25.13 ± 0.09 ^bE^	25.66 ± 0.07 ^bD^	26.33 ± 0.25 ^cB^	26.96 ± 0.08 ^bA^	26.06 ± 0.09 ^bC^
CH 0.50%	25.00 ± 0.13 ^bE^	25.46 ± 0.09 ^bC^	25.73 ± 0.09 ^dB^	26.33 ± 0.24 ^bA^	25.33 ± 0.09 ^cD^
CH 0.75%	24.06 ± 0.09 ^cE^	24.40 ± 0.16 ^cC^	24.80 ± 0.16 ^eB^	25.60 ± 0.32 ^cA^	24.33 ± 0.25 ^dD^
XG 0.1%	23.4 ± 0.28 ^dE^	24.06 ± 0.07 ^cD^	24.73 ± 0.22 ^eB^	25.53 ± 0.11 ^cA^	24.26 ± 0.24 ^dC^
XG 0.2%	22.4 ±0.20 ^eE^	22.93 ± 0.12 ^eC^	23.46 ± 0.18 ^fD^	24.46 ± 0.17 ^dA^	24.13 ± 0.09 ^dB^
XG 0.3%	21.86 ± 0.18 ^fE^	22.46 ± 0.07 ^eD^	23.21 ± 0.16 ^fC^	24.13 ± 0.04 ^dA^	23.58 ± 0.25 ^eB^
(C) Titratable acidity (%)					
CL	1.85 ± 0.12 ^hA^	1.64 ± 0.03 ^iB^	1.40 ± 0.05 ^jC^	1.15 ± 0.05 ^hD^	0.91 ± 0.06 ^aE^
AL 1%	2.01 ± 0.03 ^fA^	1.87 ± 0.08 ^fB^	1.74 ± 0.09 ^gC^	1.61 ± 0.13 ^fD^	1.47 ± 0.05 ^gE^
AL 2%	2.13 ± 0.07 ^dA^	2.04 ± 0.05 ^dB^	1.92 ± 0.03 ^dC^	1.79 ± 0.11 ^dD^	1.68 ± 0.06 ^fE^
AL 3%	2.21 ± 0.06 ^bA^	2.15 ± 0.04 ^bB^	2.04 ± 0.04 ^bC^	1.94 ± 0.03 ^aD^	1.75 ± 0.06 ^cE^
CH 0.25%	1.92 ± 0.05 ^gA^	1.77 ± 0.03 ^hB^	1.64 ± 0.03 ^iC^	1.42 ± 0.07 ^gD^	1.23 ± 0.03 ^jE^
CH 0.50%	1.92 ± 0.05 ^gA^	1.81 ± 0.03 ^gB^	1.71 ± 0.12 ^hC^	1.61 ± 0.12 ^fD^	1.45 ± 0.06 ^iE^
CH 0.75%	2.04 ± 0.04 ^fA^	1.98 ± 0.64 ^eB^	1.89 ± 0.03 ^eC^	1.81 ± 0.03 ^cD^	1.73 ± 0.05 ^dE^
XG 0.1%	2.11 ± 0.07 ^eA^	1.98 ± 0.16 ^eB^	1.83 ± 0.03 ^fC^	1.68 ± 0.07 ^eD^	1.51 ± 0.06 ^hE^
XG 0.2%	2.19 ± 0.06 ^cA^	2.09 ± 0.03 ^cB^	1.98 ± 0.05 ^cC^	1.85 ± 0.07 ^bD^	1.72 ± 0.05 ^eE^
XG 0.3%	2.26 ± 0.03 ^aA^	2.19 ± 0.12 ^aB^	2.06 ± 0.05 ^aC^	1.94 ± 0.04 ^aD^	1.81 ± 0.03 ^bE^
(D) Ascorbic acid (mg/100 g FW)					
CL	34.83 ± 0.52 ^jA^	31.16 ± 2.07 ^jB^	28.23 ± 1.37 ^jC^	23.46 ± 0.51 ^iD^	10.26 ± 1.03 ^iE^
AL 1%	37.03 ± 2.26 ^hA^	35.93 ± 0.05 ^hB^	34.46 ± 0.53 ^hC^	30.43 ± 0.89 ^hD^	18.7 ± 0.87 ^hE^
AL 2%	41.06 ± 2.26 ^fA^	39.6 ± 1.55 ^fB^	35.56 ± 0.5 ^gC^	31.53 ± 0.52 ^fD^	21.43 ± 1.03 ^fE^
AL 3%	42.16 ± 2.07 ^dA^	41.8 ± 0.05 ^dB^	36.66 ± 0.49 ^eC^	33.36 ± 1.03 ^dD^	24.63 ± 1.04 ^dE^
CH 0.25%	35.93 ± 1.86 ^iA^	33.73 ± 1.37 ^iB^	31.16 ± 0.51 ^iC^	23.1 ± 0.03 ^jD^	11.93 ± 0.51 ^jE^
CH 0.50%	40.7 ± 1.55 ^gA^	39.23 ± 1.86 ^gB^	36.03 ± 1.03 ^fC^	31.33 ± 0.89 ^gD^	21.16 ± 0.52 ^gE^
CH 0.75%	41.26 ± 2.07 ^eA^	40.16 ± 0.52 ^eB^	38.43 ± 0.52 ^dC^	33.66 ± 0.52 ^cD^	25.83 ± 0.53 ^bE^
XG 0.1%	46.46 ± 1.37 ^cA^	43.26 ± 1.03 ^cB^	40.70 ± 0.18 ^cC^	32.26 ± 2.07 ^eD^	22.21 ± 1.55 ^eE^
XG 0.2%	48.03 ± 3.62 ^bA^	47.31 ± 0.89 ^bB^	45.46 ± 0.51 ^bC^	33.73 ± 1.37 ^bD^	25.66 ± 1.37 ^cE^
XG 0.3%	52.8 ± 0.11 ^aA^	51.33 ± 1.37 ^aB^	49.5 ± 0.12 ^aC^	39.23 ± 1.03 ^aD^	32.51 ± 1.79 ^aE^

CL: control samples; AL: sodium alginate; CH: chitosan; XG: xanthan gum. Results are expressed as means ± standard deviations. Means with the same superscripts in a column are not significantly different (*p* < 0.05), as assessed by Duncan’s multiple range test.

**Table 2:** Effects of polysaccharide-based coatings and storage on total phenolic content (mg GAE/g DW), ABTS assay (% inhibition), DPPH radical scavenging activity (% inhibition) and lipid peroxidation assay (% inhibition) values.

Treatment	Days of Storage				
	2	4	6	8	10
(A) Total phenolic content					
CL	0.56 ± 0.02 ^ap^	0.63 ± 0.03 ^aq^	0.68 ± 0.03 ^ar^	0.78 ± 0.05 ^as^	0.61 ± 0.04 ^ap^
AL 1%	0.66 ± 0.05 ^fp^	0.68 ± 0.07 ^cdq^	0.79 ± 0.03 ^der^	1.24 ± 0.04 ^is^	0.79 ± 0.03 ^fr^
AL 2%	0.65 ± 0.02 ^eq^	0.68 ± 0.02 ^cdr^	0.79 ± 0.03 ^cds^	1.03 ± 0.03 ^et^	0.64 ± 0.02 ^ap^
AL 3%	0.65 ± 0.07 ^eq^	0.67 ± 0.05 ^cqr^	0.71 ± 0.02 ^ar^	0.81 ± 0.04 ^bs^	0.63 ± 0.06 ^ap^
CH 0.25%	0.57 ± 0.09 ^bp^	0.66 ± 0.03 ^bq^	0.71 ± 0.08 ^ar^	0.88 ± 0.07 ^cs^	0.63 ± 0.03 ^ap^
CH 0.50%	0.58 ± 0.05 ^bp^	0.68 ± 0.02 ^dq^	0.74 ± 0.03 ^br^	0.96 ± 0.07 ^ds^	0.66 ± 0.04 ^bp^
CH 0.75%	0.61 ± 0.04 ^cp^	0.73 ± 0.07 ^gq^	0.80 ± 0.08 ^eq^	0.98 ± 0.05 ^dr^	0.68 ± 0.09 ^dp^
XG 0.1%	0.67 ± 0.05 ^gp^	0.73 ± 0.02 ^gq^	0.79 ± 0.03 ^der^	1.22 ± 0.07 ^ht^	0.88 ± 0.08 ^gs^
XG 0.2%	0.65 ± 0.02 ^ep^	0.71 ± 0.02 ^fq^	0.78 ± 0.04 ^cder^	1.05 ± 0.03 ^ft^	0.86 ± 0.02 ^gs^
XG 0.3%	0.63 ± 0.03 ^dp^	0.69 ± 0.07 ^eq^	0.77 ± 0.02 ^cr^	0.82 ± 0.04 ^bs^	0.69 ± 0.02 ^eq^
(B) ABTS assay					
CL	62.51 ± 0.1 ^ap^	65.19 ± 0.38 ^aq^	68.35 ± 0.37 ^as^	70.11 ± 0.17 ^at^	61.01 ± 0.45 ^ap^
AL 1%	67.76 ± 0.11 ^fq^	69.13 ± 0.27 ^dq^	71.07 ± 0.10 ^cdr^	73.86 ± 0.10 ^cds^	66.59 ± 0.54 ^cp^
AL 2%	66.81 ± 0.2 ^eq^	68.06 ± 0.35 ^cr^	69.45 ± 0.10 ^bs^	72.83 ± 0.21 ^bct^	63.36 ± 0.43 ^abp^
AL 3%	66.37 ± 0.11 ^deq^	67.32 ± 0.12 ^bcq^	68.86 ± 0.10 ^abr^	70.63 ± 0.10 ^as^	62.48 ± 1.06 ^ap^
CH 0.25%	63.14 ± 0.28 ^bp^	66.21 ± 0.10 ^bq^	68.64 ± 0.27 ^ar^	72.68 ± 0.35 ^bs^	62.31 ± 0.19 ^ap^
CH 0.50%	63.36 ± 0.28 ^bp^	67.32 ± 0.11 ^bcq^	70.55 ± 0.11 ^cr^	73.42 ± 1.34 ^bcds^	64.25 ± 0.83 ^bp^
CH 0.75%	64.79 ± 0.16 ^cp^	69.53 ± 0.21 ^dr^	72.76 ± 0.10 ^es^	73.86 ± 0.37 ^cdt^	66.28 ± 0.58 ^cq^
XG 0.1%	67.84 ± 0.78 ^fp^	69.97 ± 0.10 ^dq^	71.36 ± 0.78 ^dqr^	74.30 ± 0.27 ^ds^	71.91 ± 0.83 ^er^
XG 0.2%	66.44 ± 0.81 ^dep^	68.13 ± 0.63 ^cq^	70.55 ± 0.10 ^cr^	71.07 ± 0.10 ^ar^	69.16 ± 0.31 ^dq^
XG 0.3%	65.19 ± 0.38 ^cdp^	67.25 ± 0.68 ^bq^	69.38 ± 0.35 ^br^	70.56 ± 0.31 ^ar^	65.81 ± 0.99 ^cp^
(C) DPPH radical scavenging activity					
CL	61.57 ± 0.26 ^ap^	68.81 ± 0.22 ^aq^	71.05 ± 0.25 ^ar^	78.36 ± 0.06 ^as^	62.90 ± 0.15 ^ar^
AL 1%	73.12 ± 0.26 ^fp^	75.24 ± 0.14 ^cq^	83.15 ± 0.24 ^fr^	90.89 ± 0.33 ^es^	74.76 ± 0.67 ^eq^
AL 2%	72.98 ± 0.35 ^fq^	75.19 ± 0.11 ^cr^	81.84 ± 0.20 ^ds^	87.77 ± 0.20 ^dt^	68.47 ± 0.30 ^dp^
AL 3%	72.88 ± 0.13 ^fq^	73.24 ± 0.15 ^bq^	75.29 ± 0.06 ^br^	77.88 ± 0.32 ^as^	63.08 ± 0.18 ^ap^
CH 0.25%	63.12 ± 0.23 ^bp^	72.81 ± 0.33 ^br^	75.36 ± 0.12 ^bs^	86.82 ± 0.18 ^ct^	64.54 ± 0.21 ^bq^
CH 0.50%	64.83 ± 0.21 ^cp^	75.29 ± 0.06 ^cr^	80.31 ± 0.16 ^cs^	91.30 ± 0.06 ^et^	64.78 ± 0.23 ^bq^
CH 0.75%	67.54 ± 0.10 ^dq^	79.58 ± 0.41 ^er^	85.22 ± 0.29 ^gs^	92.18 ± 0.22 ^ft^	65.11 ± 0.21 ^cp^
XG 0.1%	73.19 ± 0.10 ^fp^	79.58 ± 0.36 ^er^	83.17 ± 0.22 ^fs^	90.82 ± 0.29 ^et^	75.02 ± 0.18 ^fq^
XG 0.2%	72.86 ± 0.16 ^fp^	75.41 ± 0.05 ^cr^	82.51 ± 0.37 ^es^	88.30 ± 0.54 ^dt^	73.81 ± 0.38 ^eq^
XG 0.3%	68.81 ± 0.22 ^eq^	76.17 ± 0.24 ^dr^	80.12 ± 0.05 ^cs^	82.77 ± 0.05 ^bt^	67.88 ± 0.18 ^dp^
(D) Lipid peroxidation assay					
CL	33.49 ± 0.74 ^ap^	35.45 ± 0.37 ^ap^	37.06 ± 0.31 ^aq^	39.87 ± 0.44 ^ar^	35.09 ± 0.92 ^ap^
AL 1%	39.51 ± 0.32 ^ep^	40.17 ± 0.58 ^eq^	41.77 ± 0.74 ^eq^	43.89 ± 0.44 ^dr^	39.22 ± 0.43 ^dp^
AL 2%	38.31 ± 1.09 ^dp^	39.96 ± 0.12 ^eq^	40.57 ± 0.42 ^dqr^	41.59 ± 0.74 ^bcr^	37.35 ± 0.31 ^bp^
AL 3%	35.54 ± 0.25 ^bp^	37.65 ± 0.39 ^cq^	39.01 ± 0.43 ^cr^	41.37 ± 0.18 ^bcs^	36.09 ± 0.14 ^ap^
CH 0.25%	34.63 ± 0.21 ^ap^	36.34 ± 0.25 ^bq^	38.32 ± 0.35 ^br^	40.16 ± 0.58 ^as^	37.60 ± 0.18 ^br^
CH 0.50%	35.34 ± 0.26 ^bp^	37.30 ± 0.23 ^cq^	39.56 ± 0.23 ^cr^	41.08 ± 0.37 ^abs^	38.61 ± 0.32 ^cdr^
CH 0.75%	35.54 ± 0.25 ^bp^	38.56 ± 0.11 ^dq^	41.62 ± 0.42 ^er^	42.13 ± 0.37 ^bcr^	38.96 ± 0.21 ^dq^
XG 0.1%	39.81 ± 0.37 ^ep^	40.42 ± 0.21 ^eq^	42.87 ± 0.46 ^fr^	44.63 ± 0.86 ^er^	39.86 ± 0.43 ^dp^
XG 0.2%	37.15 ± 0.31 ^cp^	38.96 ± 0.46 ^dq^	40.92 ± 0.75 ^der^	42.43 ± 0.93 ^bcs^	37.85 ± 0.36 ^bcpq^
XG 0.3%	35.39 ± 0.43 ^bp^	37.55 ± 0.44 ^cq^	38.56 ± 0.58 ^aq^	41.72 ± 0.51 ^bcr^	36.34 ± 0.32 ^ap^

ABTS; 2,2′-azino-bis(3-ethylbenzothiazoline-6-sulfonic acid and DPPH; 2,2-diphenyl-1-picryl-hydrazyl; CL: control samples; AL: sodium alginate; CH: chitosan; XG: xanthan gum. Results are expressed as means ± standard deviations. Means with the same superscripts in a column are not significantly different (*p* < 0.05), as assessed by Duncan’s multiple range test.

**Table 3:** Effects of polysaccharide-based coatings and storage on neochlorogenic acid, chlorogenic acid, ellagic acid and epicatechin levels (mg/Kg DW).

Treatment	Days of Storage				
	2	4	6	8	10
(A) Neochlorogenic acid					
CL	10.92 ± 0.13 ^prs^	13.67 ± 0.31 ^mnq^	16.91 ± 0.36 ^jkl^	22.52 ± 0.44 ^df^	19.37 ± 0.24 ^gj^
AL 1%	11.29 ± 0.23 ^opq^	16.22 ± 0.29 ^klm^	19.37 ± 0.21 ^ghi^	25.16 ± 0.36 ^cd^	22.16 ± 0.31 ^ef^
AL 2%	13.96 ± 0.43 ^mno^	15.11 ± 0.27 ^lmn^	18.06 ± 0.25 ^ik^	24.16 ± 0.21 ^cde^	28.06 ± 0.35 ^ab^
AL 3%	10.67 ± 0.44 ^qstuv^	12.15 ± 0.16 ^opt^	14.82 ± 0.16 ^lmn^	18.32 ± 0.26 ^hijk^	21.61 ± 0.31 ^efg^
CH 0.25%	6.84 ± 0.34 ^xz^	8.37 ± 0.26 ^uxy^	10.38 ± 0.14 ^stuv^	22.42 ± 0.37 ^df^	29.42 ± 0.25 ^a^
CH 0.50%	5.58 ± 0.32 ^z^	7.19 ± 0.16 ^wz^	9.36 ± 0.25 ^tux^	17.61 ± 0.16 ^ijkl^	22.82 ± 0.23 ^cdef^
CH 0.75%	6.28 ± 0.24 ^yz^	7.40 ± 0.18 ^wxyz^	8.12 ± 0.08 ^vwx^	16.17 ± 0.31 ^klm^	21.23 ± 0.22 ^efh^
XG 0.1%	11.19 ± 0.22 ^optu^	12.12 ± 0.20 ^opt^	13.42 ± 0.27 ^opqr^	20.19 ± 0.28 ^fi^	25.64 ± 0.17 ^bc^
XG 0.2%	8.981 ± 0.53 ^uxy^	10.69 ± 0.26 ^psv^	12.62 ± 0.14 ^nqrs^	16.22 ± 0.24 ^klm^	20.39 ± 0.27 ^fgi^
XG 0.3%	7.141 ± 0.33 ^xyz^	10.11 ± 0.12 ^stw^	13.67 ± 0.38 ^mp^	17.56 ± 0.30 ^ijl^	21.34 ± 0.43 ^efg^
(B) Chlorogenic acid					
CL	18.31 ± 0.43 ^pqr^	19.63 ± 0.35 ^ipq^	21.69 ± 0.18 ^deijk^	25.2 ± 0.31 ^abc^	23.11 ± 0.11 ^cdef^
AL 1%	20.11 ± 0.16 ^hpq^	21.2 ± 0.21 ^fgn^	22.95 ± 0.16 ^cdg^	26.71 ± 0.23 ^a^	25.18 ± 0.27 ^abc^
AL 2%	18.92 ± 0.24 ^1rs^	19.11 ± 0.12 ^lms^	21.11 ± 0.15 ^fmn^	25.54 ± 0.27 ^ab^	23.16 ± 0.32 ^cdef^
AL 3%	19.66 ± 0.31 ^ijpq^	19.69 ± 0.27 ^ipq^	19.92 ± 0.08 ^ipq^	23.18 ± 0.17 ^cde^	21.33 ± 0.16 ^efjk^
CH 0.25%	16.07 ± 0.28 ^tu^	18.31 ± 0.18 ^prst^	20.65 ± 0.41 ^gop^	26.45 ± 0.11 ^a^	23.82 ± 0.27 ^bcd^
CH 0.50%	16.33 ± 0.11 ^tu^	17.05 ± 0.07 ^stu^	17.85 ± 0.27 ^qrst^	21.92 ± 0.34 ^deh^	19.21 ± 0.15 ^qrs^
CH 0.75%	15.15 ± 0.15 ^u^	16.36 ± 0.07 ^tu^	17.01 ± 0.39 ^stu^	23.68 ± 0.30 ^bde^	19.39 ± 0.32 ^kqr^
XG 0.1%	17.33 ± 0.42 ^rst^	18.21 ± 0.11 ^qrst^	19.61 ± 0.18 ^ijpqr^	23.77 ± 0.13 ^bcd^	21.74 ± 0.27 ^dhij^
XG 0.2%	19.14 ± 0.20 ^ls^	19.31 ± 0.10 ^lqrs^	19.51 ± 0.06 ^jkqr^	22.61 ± 0.22 ^deh^	18.38 ± 0.44 ^pqst^
XG 0.3%	17.14 ± 0.34 ^rstu^	18.14 ± 0.18 ^lmrs^	18.83 ± 0.22 ^nrs^	21.25 ± 0.21 ^glm^	18.96 ± 0.58 ^qrs^
(C) Ellagic acid					
CL	0.42 ± 0.02 ^opu^	0.43 ± 0.01 ^opuv^	0.46 ± 0.03 ^out^	1.21 ± 0.03 ^def^	1.06 ± 0.01 ^efgh^
AL 1%	0.24 ± 0.03 ^uvw^	0.26 ± 0.03 ^tuvw^	0.29 ± 0.02 ^stuv^	0.59 ± 0.01 ^mr^	0.45 ± 0.01 ^ov^
AL 2%	0.76 ± 0.02 ^jkl^	0.95 ± 0.05 ^ghi^	1.33 ± 0.01 ^cd^	1.55 ± 0.02 ^c^	1.41 ± 0.03 ^cd^
AL 3%	0.22 ± 0.01 ^uvw^	0.22 ± 0.04 ^uvw^	0.25 ± 0.01 ^uvw^	0.29 ± 0.03 ^sw^	0.27 ± 0.02 ^tuv^
CH 0.25%	0.29 ± 0.03 ^stw^	0.37 ± 0.03 ^qrs^	0.63 ± 0.03 ^klm^	1.21 ± 0.02 ^def^	0.97 ± 0.02 ^f^
CH 0.50%	0.73 ± 0.04 ^jn^	0.86 ± 0.01 ^hijk^	0.98 ± 0.04 ^fij^	1.52 ± 0.02 ^c^	2.62 ± 0.01 ^b^
CH 0.75%	1.05 ± 0.03 ^efi^	1.26 ± 0.02 ^de^	1.51 ± 0.01 ^c^	2.61 ± 0.01 ^b^	3.71 ± 0.02 ^a^
XG 0.1%	0.23 ± 0.01 ^uvw^	0.41 ± 0.01 ^opv^	0.56 ± 0.02 ^lmq^	0.83 ± 0.03 ^ijk^	0.78 ± 0.03 ^jkl^
XG 0.2%	0.41 ± 0.07 ^pvw^	0.49 ± 0.02 ^nos^	0.53 ± 0.01 ^lmno^	0.67 ± 0.02 ^klo^	0.51 ± 0.04 ^mno^
XG 0.3%	0.18 ± 0.02 ^w^	0.19 ± 0.03 ^vw^	0.21 ± 0.01 ^vw^	0.31 ± 0.03 ^rsw^	0.29 ± 0.02 ^stuw^
(D) Epicatechin					
CL	31.12 ± 0.21 ^hi^	32.61 ± 0.21 ^ghi^	35.96 ± 0.42 ^efg^	38.18 ± 0.27 ^e^	34.51 ± 0.11 ^fgh^
AL 1%	48.70 ± 0.32 ^c^	52.33 ± 0.31 ^b^	59.50 ± 0.58 ^a^	62.31 ± 0.24 ^a^	55.58 ± 0.59 ^b^
AL 2%	36.12 ± 0.25 ^ef^	37.31 ± 0.34 ^ef^	41.68 ± 0.39 ^d^	45.23 ± 0.39 ^c^	38.25 ± 0.47 ^de^
AL 3%	17.31 ± 0.11 ^vwxy^	22.79 ± 0.26 ^qu^	26.17 ± 0.22 ^mnq^	29.52 ± 0.27 ^ijkl^	25.33 ± 0.25 ^mr^
CH 0.25%	23.22 ± 0.27 ^pqt^	25.61 ± 0.16 ^mnr^	26.33 ± 0.28 ^mno^	28.51 ± 0.21 ^jlm^	23.67 ± 0.19 ^ops^
CH 0.50%	16.15 ± 0.12 ^xyz^	18.18 ± 0.30 ^vwxy^	22.22 ± 0.19 ^rstu^	24.76 ± 0.29 ^nor^	23.57 ± 0.22 ^ops^
CH 0.75%	16.86 ± 0.17 ^vwz^	20.18 ± 0.27 ^stuv^	23.36 ± 0.11 ^pqrs^	26.82 ± 0.30 ^klm^	24.66 ± 0.27 ^nqr^
XG 0.1%	16.76 ± 0.09 ^wxyz^	19.62 ± 0.31 ^uvwx^	23.99 ± 0.18 ^opqr^	26.72 ± 0.25 ^lmp^	23.77 ± 0.22 ^ops^
XG 0.2%	23.93 ± 0.24 ^or^	25.52 ± 0.43 ^mnor^	27.16 ± 0.23 ^klmn^	29.66 ± 0.30 ^ijk^	28.35 ± 0.24 ^jm^
XG 0.3%	13.81 ± 0.13 ^z^	15.72 ± 0.20 ^yz^	17.72 ± 0.09 ^vwxy^	19.98 ± 0.18 ^tuv^	18.13 ± 0.19 ^vw^

CL: control samples; AL: sodium alginate; CH: chitosan; XG: xanthan gum. Results are expressed as means ± standard deviations. Means with the same superscripts in a column are not significantly different (*p* < 0.05), as assessed by Duncan’s multiple range test.

**Table 4:** Effects of polysaccharide-based coatings and storage on phloridzin, kaempferol, Quercetin-3-glucoside and procyanidin b_2_ levels (mg/Kg DW).

Treatment	Days of Storage				
	2	4	6	8	10
(A) Phloridzin					
CL	5.14 ± 0.12 ^pqr^	5.49 ± 0.26 ^cdr^	6.25 ± 0.22 ^fgh^	6.61 ± 0.11 ^cgi^	6.08 ± 0.28 ^dfg^
AL 1%	4.35 ± 0.21 ^rst^	4.92 ± 0.21 ^efi^	5.29 ± 0.14 ^fkl^	5.95 ± 0.22 ^def^	5.62 ± 0.25 ^ghi^
AL 2%	5.37 ± 0.18 ^ls^	8.31 ± 0.32 ^frs^	18.17 ± 0.4 ^lmn^	12.21 ± 0.34 ^br^	6.14 ± 0.17 ^acq^
AL 3%	2.62 ± 0.11 ^tu^	2.81 ± 0.31 ^ifij^	3.90 ± 0.27 ^dfji^	4.41 ± 0.56 ^mno^	3.40 ± 0.22 ^br^
CH 0.25%	2.80 ± 0.14 ^hpq^	3.61 ± 0.14 ^cgik^	4.19 ± 0.23 ^cfi^	4.65 ± 0.12 ^acn^	4.37 ± 0.36 ^cf^
CH 0.50%	4.41 ± 0.2^1^	4.81 ± 0.18 ^dgj^	5.25 ± 0.33 ^mn^	5.71 ± 0.36 ^fop^	2.62 ± 0.02 ^cbh^
CH 0.75%	4.39 ± 0.11 ^irs^	4.90 ± 0.26 ^dhk^	5.73 ± 0.16 ^prs^	5.95 ± 0.12 ^cd^	4.01 ± 0.11 ^djk^
XG 0.1%	2.13 ± 0.30 ^cdr^	2.51 ± 0.15 ^mn^	2.74 ± 0.49 ^cdg^	3.61 ± 0.19 ^bfh^	6.28 ± 0.09 ^ijm^
XG 0.2%	3.01 ± 0.22 ^tu^	4.21 ± 0.12 ^cgh^	5.05 ± 0.20 ^bdi^	5.51 ± 0.32 ^ckl^	2.86 ± 0.05 ^jk^
XG 0.3%	2.4 ± 0.26 ^bcg^	2.92 ± 0.16 ^def^	3.90 ± 0.24 ^cfi^	5.71 ± 0.18 ^klm^	7.62 ± 0.07 ^mo^
(B) Kaempferol					
CL	0.91 ± 0.03 ^at^	0.92 ± 0.05 ^cp^	1.20 ± 0.08 ^ap^	1.61 ± 0.09 ^fgh^	1.50 ± 0.05 ^djk^
AL 1%	0.96 ± 0.02 ^bp^	1.15 ± 0.02 ^abrs^	1.23 ± 0.09 ^cp^	1.56 ± 0.02 ^bq^	1.45 ± 0.07 ^rt^
AL 2%	0.51 ± 0.04 ^det^	0.57 ± 0.02 ^des^	0.64 ± 0.06 ^ep^	0.71 ± 0.01 ^cp^	0.69 ± 0.02 ^epq^
AL 3%	0.16 ± 0.02 ^fs^	0.16 ± 0.05 ^bfs^	0.16 ± 0.04 ^be^	0.19 ± 0.05 ^cde^	0.19 ± 0.01 ^ij^
CH 0.25%	0.11 ± 0.08 ^act^	0.12 ± 0.05 ^bs^	0.19 ± 0.02 ^cde^	0.21 ± 0.09 ^efg^	0.19 ± 0.04 ^pq^
CH 0.50%	0.13 ± 0.03 ^at^	0.15 ± 0.02 ^acr^	0.16 ± 0.05 ^eq^	0.19 ± 0.06 ^cd^	0.27 ± 0.02 ^fgh^
CH 0.75%	0.16 ± 0.07 ^bcs^	0.18 ± 0.09 ^eds^	0.20 ± 0.07 ^deq^	0.22 ± 0.06 ^ijk^	0.11 ± 0.02 ^brs^
XG 0.1%	0.27 ± 0.01 ^ct^	0.28 ± 0.04 ^cfr^	0.21 ± 0.06 ^ecp^	0.33 ± 0.08 ^de^	0.18 ± 0.04 ^mn^
XG 0.2%	0.16 ± 0.06 ^des^	0.19 ± 0.09 ^ders^	0.24 ± 0.09 ^ebd^	0.25 ± 0.06 ^lm^	0.21 ± 0.01 ^efg^
XG 0.3%	0.21 ± 0.03 ^fs^	0.23 ± 0.04 ^frs^	0.41 ± 0.06 ^cdp^	0.42 ± 0.01 ^bcd^	0.42 ± 0.05 ^det^
(C) Quercetin-3-glucoside					
CL	1.34 ± 0.09 ^dfh^	1.41 ± 0.09 ^ikm^	1.43 ± 0.03 ^jmn^	1.52 ± 0.08 ^efr^	1.31 ± 0.04 ^df^
AL 1%	2.34 ± 0.02 ^bfg^	2.51 ± 0.04 ^lm^	3.74 ± 0.08 ^cfg^	3.82 ± 0.07 ^bcg^	3.71 ± 0.08 ^cjk^
AL 2%	2.65 ± 0.06 ^ad^	2.81 ± 0.05 ^cdm^	3.41 ± 0.06 ^hi^	3.22 ± 0.01 ^abp^	1.79 ± 0.06 ^pq^
AL 3%	2.31 ± 0.07 ^cfg^	2.52 ± 0.02 ^efg^	2.81 ± 0.08 ^afg^	2.91 ± 0.03 ^cde^	0.43 ± 0.09 ^ade^
CH 0.25%	2.71 ± 0.01 ^acd^	2.95 ± 0.01 ^afe^	3.17 ± 0.06 ^pqr^	3.21 ± 0.08 ^fg^	4.11 ± 0.03 ^cdf^
CH 0.50%	2.08 ± 0.04 ^kqr^	2.19 ± 0.05 ^dt^	2.32 ± 0.09 ^ef^	2.41 ± 0.05 ^bce^	2.26 ± 0.08 ^adg^
CH 0.75%	3.14 ± 0.01 ^jk^	3.21 ± 0.02 ^cdr^	3.29 ± 0.03 ^bfh^	3.32 ± 0.05 ^lmn^	6.32 ± 0.08 ^cf^
XG 0.1%	3.61 ± 0.05 ^bc^	3.96 ± 0.05 ^adh^	3.81 ± 0.02 ^fi^	4.98 ± 0.03 ^cdg^	2.96 ± 0.09 ^mn^
XG 0.2%	2.82 ± 0.08 ^lm^	3.19 ± 0.06 ^pqr^	3.46 ± 0.01 ^ikl^	3.94 ± 0.08 ^kl^	2.33 ± 0.06 ^ij^
XG 0.3%	2.39 ± 0.06 ^bef^	2.91 ± 0.09 ^cde^	3.28 ± 0.07 ^fgr^	3.85 ± 0.07 ^bq^	1.20 ± 0.02 ^ch^
(D) Procyanidin b_2_					
CL	6.12 ± 0.18 ^cfh^	6.22 ± 0.17 ^bk^	6.35 ± 0.24 ^de^	6.67 ± 0.22 ^an^	6.86 ± 0.16 ^efj^
AL 1%	9.12 ± 0.12 ^ef^	9.51 ± 0.23 ^acg^	10.32 ± 0.18 ^fj^	11.79 ± 0.14 ^cfn^	11.01 ± 0.12 ^deh^
AL 2%	9.16 ± 0.24 ^bck^	9.25 ± 0.12 ^cd^	9.38 ± 0.16 ^ach^	10.72 ± 0.18 ^cd^	10.61 ± 0.34 ^df^
AL 3%	6.66 ± 0.22 ^gh^	6.75 ± 0.24 ^fgh^	7.39 ± 0.25 ^dk^	7.88 ± 0.29 ^dgn^	7.58 ± 0.26 ^bcf^
CH 0.25%	6.02 ± 0.32 ^efk^	6.21 ± 0.12 ^mn^	6.51 ± 0.22 ^bfi^	6.82 ± 0.15 ^ach^	6.75 ± 0.12 ^achi^
CH 0.50%	6.26 ± 0.25 ^cd^	6.62 ± 0.22 ^be^	6.70 ± 0.14 ^efn^	6.91 ± 0.14 ^bfh^	6.62 ± 0.15 ^jkl^
CH 0.75%	7.20 ± 0.19 ^ac^	7.52 ± 0.20 ^bdg^	7.75 ± 0.51 ^hi^	7.92 ± 0.22 ^ikl^	7.74 ± 0.17 ^cfj^
XG 0.1%	6.86 ± 0.14 ^abf^	6.61 ± 0.17 ^bch^	8.91 ± 0.42 ^ehi^	7.21 ± 0.17 ^fgi^	7.41 ± 0.11 ^dmn^
XG 0.2%	6.69 ± 0.34 ^dfg^	6.81 ± 0.24 ^cgj^	8.70 ± 0.45 ^acn^	7.25 ± 0.13 ^klmn^	12.77 ± 0.26 ^chi^
XG 0.3%	6.11 ± 0.40 ^kl^	6.31 ± 0.19d ^ef^	8.82 ± 0.33 ^bc^	7.36 ± 0.18 ^cghi^	9.27 ± 0.19 ^mn^

CL: control samples; AL: sodium alginate; CH: chitosan; XG: xanthan gum. Results are expressed as means ± standard deviations. Means with the same superscripts in a column are not significantly different (*p* < 0.05), as assessed by Duncan’s multiple range test.

## Data Availability

Not applicable.
